# The risk of peripartum cardiomyopathy among pediatric, adolescent, and young adult cancer patients exposed to doxorubicin: an opinion article

**DOI:** 10.3389/fonc.2024.1395465

**Published:** 2024-11-12

**Authors:** Christina Carfagnini, Simon Bechara, Manasa Kandula

**Affiliations:** Department of Internal Medicine, University of Illinois College of Medicine Peoria, Peoria, IL, United States

**Keywords:** cardiotoxicity, anthracyclines, doxorubicin, pregnancy, peripartum cardiomyopathy

## 1 Introduction

The impact of cardiotoxic chemotherapy on female pediatric cancer survivors as they reach the child-bearing age has not been well researched. In the United States, the incidence of malignancy among childhood, adolescent, and young adult (AYA) patients has been increasing since the 1970s (1, 2). However, the 5-year survival rates also increased for this population (3, 4), suggesting a growing population of female cancer survivors reaching reproductive potential. The most common malignancies among pediatric and AYA females include leukemias, Hodgkin’s lymphomas, thyroid carcinoma, and breast carcinoma (4). These malignancies are treated with doxorubicin, a cardiotoxic anthracycline. Doxorubicin causes cardiac injury by increased free-radical generation in the mitochondria, decreased topoisomerase-2-beta activation leading to increased p53 and decreased STAT activity, and decreased ErbB signaling leading to decreased VEGF expression (5). These molecular alterations lead to development of fibrous tissue in the myocardium, which causes increased thickness of the left ventricular (LV) wall compared with overall bodily growth in children. Such changes diminish functional reserve, making individuals more susceptible to acute cardiac deterioration when exposed to additional stress (6). Therefore, the ability to accommodate increased maternal blood volume and cardiac output during pregnancy in those with doxorubicin exposure needs further investigation.

## 2 Current literature assessing the risk of peripartum systolic dysfunction among those exposed to anthracyclines during childhood

Previous literature reviews have discussed the risk of peripartum heart failure among patients exposed to cardiotoxic chemotherapy during childhood (6, 7). A meta-analysis aimed to determine the incidence of LV systolic dysfunction in pregnancy until 12 months postpartum among patients exposed to doxorubicin during childhood (7). This meta-analysis found that peripartum heart failure affected 1.7% (95% CI: 0.9% to 2.7%) of the study population. Although this incidence of systolic dysfunction in this meta-analysis appears low, the risk of peripartum cardiomyopathy among AYAs exposed to doxorubicin is 55 times greater than the general population (95% CI 6.6–192.0) (8). Conclusions from this meta-analysis are limited by small sample sizes and heterogeneity across study designs. Three different definitions for LV systolic dysfunction were used across five individual studies (7). The included studies also varied regarding the mean dose of doxorubicin and time from chemotherapy exposure to pregnancy, which positively correlate with peripartum cardiomyopathy (6, 7). For this reason, well-controlled studies applying a consistent measure of LV systolic function and stratifying patients into different groups based on anthracycline dose administered are needed.

Future studies should focus on identifying those at risk of poor maternal health outcomes after doxorubicin exposure. The odds of pregnancy-related heart failure were 47.4 (95% CI 17.9–125.8) times greater among patients with a decrease in LV ejection fraction (EF) before pregnancy when compared with cancer patients with similar exposures and preserved LV EF (7). However, anthracyclines often lead to subclinical cardiac dysfunction, with abnormal echocardiographic findings present in only 25%–50% of asymptomatic cancer survivors (6). An algorithm for monitoring cardiac function throughout pregnancy in patients exposed to anthracycline during childhood considered three risk factors for peripartum systolic dysfunction among those with a normal LV EF. These include cancer diagnosis before age 7, anthracycline dose greater than 250 mg/m^2^, and more than 15 years between treatment and pregnancy (6). This algorithm aligns with findings from Nolan and colleagues (7), although exact values in this algorithm have yet to be validated. Identifying those with subclinical cardiotoxicity would inform patients’ decisions regarding reproduction and provide an opportunity to intervene before clinical symptoms occur. While exposure to heart failure medications effectively reduces cardiac damage from doxorubicin (9), the teratogenicity of these medications may limit such opportunities during pregnancy.

## 3 Cardiotoxicity monitoring tools yet to be applied to pregnant populations

Studies validating a comprehensive risk assessment score for cardiovascular outcomes in pregnant cancer survivors previously exposed to anthracyclines, similar to those that exist for the general population, are lacking. There are several scoring systems used to stratify the risk of cardiotoxicity in patients who are planning to receive cardiotoxic chemotherapies, which are summarized in **Table 1** (10–12). These scores focus on patient-related risk factors to assess the risk of cardiotoxicity, with the HFA-ICOS Risk Assessment Tool being the most recently published (11). This review focuses on the CardTox score which includes prognostic markers, such as global longitudinal strain (GLS) less than 20% or pro-Brain Natriuretic Peptide (pro-BNP) greater than 400 pg/dl that other risk assessments do not (13). A CardTox score greater than 6 has 100% sensitivity and 84.2% specificity for chemotherapy-induced cardiotoxicity (13). All of these scoring systems were developed using a mixed sex cohort with advanced age, which is not generalizable to pregnant patients. For example, no score considers family history of cardiomyopathy or genetic variant-associated cardiomyopathy. Family history may be especially relevant when assessing the risk of peripartum cardiomyopathy, which shares similar genetic variants as idiopathic dilated cardiomyopathy (14), as this population is generally younger with less cardiovascular risk parameters. Interestingly, Nolan and colleagues found that current use of cardiovascular medications was associated with peripartum cardiomyopathy among pregnant cancer survivors exposed to anthracyclines with a normal LV EF before pregnancy (7). Given the cardioprotective effects of heart failure medications, these results may reflect a greater risk of peripartum cardiomyopathy among patients with increased cardiovascular risk parameters as opposed to medication exposure.

**Table 1:** Summary of patient-related criteria used in cardiotoxicity risk assessments.

Scoring system	Citation	Criteria
Heart Failure Association International Cardio-Oncology Society (HFA-ICOS) Risk Assessment Tool	Samuel et. al., 2023 (11)	Female sex Age >65 Hypertension Smoking Obesity Hyperlipidemia Diabetes mellitus History of cardiac disease Prior history of mediastinal radiation Prior history of anthracyclines or trastuzumab
Mayo Clinic Scoring System	Regino et. al., 2022 (10)	Heart disease or heart failure Coronary artery disease or equivalent Arterial hypertension Diabetes mellitus Prior history of anthracycline use Prior history of chest radiation Female sex 65 years
Cardiotoxicity (CardTox) Score	Ozturk et. al., 2021 (13)	Age >60 years Coronary artery disease Hypertension Diabetes mellitus NT-pro-BNP LV-EF <50% or LV-GLS <20%
Cardiotoxicity Risk Score (CRS)	Perez et. al., 2019 (12)	Hypertension Coronary artery disease Heart failure Age <15 years or >65 years Female sex Prior history of anthracycline exposure Prior history of chest radiation

NT-Pro-BNP, N-terminal pro-brain natriuretic peptide; LV, left ventricle; EF, ejection fraction.

The CardTox score also includes prognostic biomarkers, such as pro-BNP, although it is unclear if these markers can be generalized to a pregnant population with anthracycline exposure during childhood. A study of 29 pediatric acute lymphoid leukemia patients treated with anthracyclines found serum BNP significantly correlated with decreased LV EF 1 month after treatment (15). As such, future studies could investigate a threshold for BNP or pro-BNP elevation shortly after anthracycline exposure in pediatric patients to predict the risk of peripartum heart failure prior to pregnancy. Physiological increases in serum BNP during pregnancy would make it difficult to use this biomarker to assess the risk of peripartum heart failure during pregnancy in those exposed to anthracyclines. One study found that a BNP <100 pg/ml had a negative predicative value of 100% for identifying major adverse cardiac events in pregnant women with structural heart disease (16). However, it is unclear if this value can be applied to those with cardiotoxicity from anthracyclines. Interestingly, troponin has also been shown to have a negative predictive value of 99% for major adverse cardiac events in adults exposed to anthracyclines (9). However, these findings may not apply to pediatric patients, as increased troponin after anthracycline exposure in children with acute lymphoid leukemia was not significantly related to decreased LV EF after treatment (15).

In addition, the CardTox score includes GLS, a cardiotoxic prognostic factor that has not been studied in the pregnant population. GLS refers to the relative change in length and thickness of the left ventricular myocardium (17). GLS identified cardiac dysfunction among 32.1% of cancer survivors with a preserved LV EF, whereas 5.8% of cancer survivors had an LV EF less than 50% (18). It is important to use a sensitive predictive measure of peripartum cardiomyopathy because of the low incidence and high mortality associated with this condition (19). For this reason, several professional organizations recommend using GLS to screen for cardiotoxicity in patients treated with anthracyclines (20). However, GLS-guided cardiac monitoring among patients treated with anthracyclines has been critiqued for leading to delays or discontinuation of cancer treatment without any significant change in systolic function at 1 year of follow-up when compared with EF-guided cardiac surveillance (21). The risk of peripartum cardiomyopathy among pediatric and AYA patients exposed to doxorubicin with reduced GLS has yet to be studied. Therefore, it is unclear if using a subclinical measure of cardiac dysfunction would effectively identify high-risk patients or inappropriately deter female cancer survivors from childbearing.

Extrapolating existing cardiotoxicity risk assessments to this population may not be straightforward. A unique risk assessment score, including the age of anthracycline exposure and time from diagnosis to pregnancy, along with the above described parameters is needed (6).

## 4 A call to action

Further research is needed to inform cancer survivors regarding reproduction, but there are several limitations to completing these future studies. Previous studies investigating the risk of heart failure during pregnancy among those exposed to doxorubicin used retrospective data (7). However, it is difficult to identify cases given the low incidence of peripartum cardiomyopathy, especially in the context of those exposed to chemotherapy. Therefore, multicenter or national databases are needed to obtain a sample with sufficient statistical power. While prospectively following cancer patients after doxorubicin exposure would yield a higher-quality study, such data would rely on the complex transition from pediatric to adult care. In addition, the interdisciplinary nature of this research question would be best addressed through collaboration across cardiology, oncology, and obstetrics researchers. Collaboration across these clinical departments provides the opportunity to pool resources and funding to overcome the previously described challenges.

In conclusion, the growing population of pediatric cancer survivors reaching the child-bearing age requires improved guidelines to inform decisions regarding reproduction. While current research has found an increased risk of peripartum cardiomyopathy among those with chemotherapy-related decrease in LV EF, the risk of cardiovascular outcomes in patients with preserved LV EF is unclear. Future research should focus on the risk of peripartum cardiomyopathy among patients exposed to doxorubicin with family history of cardiomyopathy, reduced GLS or other biomarkers, and developing comprehensive risk assessment scores. Such information would inform cancer survivors’ decisions regarding reproduction and provide the opportunity to mitigate poor cardiovascular outcomes during pregnancy in young female cancer survivors with subclinical cardiac toxicity.
